# Supplementary material for: MetaRibo-Seq measures translation in microbiomes
Source: Nat Commun. 2020 Jun 29;11:3268. doi: 10.1038/s41467-020-17081-z (PMC7324362; doi:10.1038/s41467-020-17081-z)
Supplement: Supplementary file 10 — Supplementary Data 7 [file 41467_2020_17081_MOESM10_ESM.zip › File2/Confidence_High_Taxonomy/83097_out.krona.html]

Javascript must be enabled to view this page.

members
magnitude
magnitudeUnassigned
count
unassigned
taxon
rank

83097\_out

91

2
superkingdom
91

91
phylum
1239

186801
class
91

186802

SRS893170\_contig\_number\_contig-100\_12256.12257SRS893187\_contig\_number\_2165
91
order
2

186803
49
family

1898203

SRS011302\_contig\_number\_contig-100\_7950.49065SRS013098\_contig\_number\_contig-100\_11771.160418SRS013476\_contig\_number\_contig-100\_592.104986SRS013951\_contig\_number\_contig-100\_6128.6128SRS013965\_contig\_number\_20863SRS014313\_contig\_number\_4817SRS014855\_contig\_number\_3390SRS014923\_contig\_number\_25879SRS015065\_contig\_number\_3860SRS015217\_contig\_number\_7651SRS015578\_contig\_number\_33486SRS015579\_contig\_number\_16203SRS016095\_contig\_number\_14097SRS016989\_contig\_number\_15450SRS017821\_contig\_number\_18638SRS018427\_contig\_number\_24072SRS018836\_contig\_number\_contig-100\_4502.95057SRS019161\_contig\_number\_29657SRS019267\_contig\_number\_contig-100\_1541.54355SRS020869\_contig\_number\_contig-100\_12677.137676SRS021948\_contig\_number\_contig-100\_71.149461SRS022609\_contig\_number\_47576SRS024492\_contig\_number\_contig-100\_8084.8085SRS042966\_contig\_number\_18481SRS045004\_contig\_number\_contig-100\_1804.159622SRS049900\_contig\_number\_contig-100\_1177.122195SRS050941\_contig\_number\_6417SRS051882\_contig\_number\_5038SRS054905\_contig\_number\_9311SRS063040\_contig\_number\_13355SRS065504\_contig\_number\_contig-100\_1141.178237SRS075984\_contig\_number\_contig-100\_3173.137684SRS077502\_contig\_number\_526SRS078242\_contig\_number\_contig-100\_17967.55588SRS104084\_contig\_number\_2849SRS1041033\_contig\_number\_contig-100\_798.138443SRS1041092\_contig\_number\_contig-100\_2979.39409SRS104485\_contig\_number\_1133SRS142923\_contig\_number\_contig-100\_9332.58783SRS143372\_contig\_number\_contig-100\_1993.64232SRS143466\_contig\_number\_7313SRS143523\_contig\_number\_24172SRS143895\_contig\_number\_45563SRS144362\_contig\_number\_15890SRS146813\_contig\_number\_contig-100\_7915.93663SRS148817\_contig\_number\_contig-100\_4436.83903SRS893270\_contig\_number\_20772SRS971275\_contig\_number\_32983
species
48

1
genus
1506553

1
species
1946695

SRS013687\_contig\_number\_16381

38
family
31979


SRS144753\_contig\_number\_contig-100\_132.133
1
38
genus
1485

1
species
2293015

SRS098644\_contig\_number\_11589

22
species

SRS011134\_contig\_number\_48794SRS014979\_contig\_number\_33717SRS015431\_contig\_number\_72224SRS015663\_contig\_number\_18266SRS015854\_contig\_number\_21922SRS019068\_contig\_number\_72369SRS022071\_contig\_number\_28707SRS024132\_contig\_number\_20253SRS051031\_contig\_number\_35434SRS075773\_contig\_number\_2301SRS075821\_contig\_number\_1407SRS076929\_contig\_number\_25132SRS077552\_contig\_number\_28448SRS097889\_contig\_number\_36972SRS1041038\_contig\_number\_8470SRS105153\_contig\_number\_29964SRS1055022\_contig\_number\_10508SRS142503\_contig\_number\_16568SRS144135\_contig\_number\_26569SRS147766\_contig\_number\_17688SRS148159\_contig\_number\_48029SRS149181\_contig\_number\_8376
1896990

species
4

SRS024075\_contig\_number\_12252SRS049773\_contig\_number\_17921SRS063985\_contig\_number\_14245SRS148424\_contig\_number\_30219
1262828


SRS014613\_contig\_number\_18962SRS015694\_contig\_number\_19328SRS018623\_contig\_number\_3627SRS047014\_contig\_number\_27705SRS052697\_contig\_number\_27751SRS053335\_contig\_number\_7936SRS076756\_contig\_number\_17254SRS1041140\_contig\_number\_12254SRS143780\_contig\_number\_22320SRS147977\_contig\_number\_11808
2293016
species
10

2
family
541000

1263
genus
2

1
species
40518

SRS064276\_contig\_number\_4479

1
species
1160721

SRS077194\_contig\_number\_1432
